# Efficiency of Blood Utilization in Elective Neurosurgery Patients and Development of a Maximum Surgical Blood Order Schedule

**DOI:** 10.7759/cureus.117004

**Published:** 2026-09-27

**Authors:** Zanib Javed, Malaika Siddiqui, Fizzah Bari, Altaf Ali Laghari, Nasr Hussain, Muhammad Ehsan Bari

**Affiliations:** 1 Surgery, Aga Khan University Hospital, Karachi, PAK; 2 Neurosurgery, Aga Khan University Hospital, Karachi, PAK; 3 Neurosurgery, Ziauddin University, Karachi, PAK

**Keywords:** cross match transfusion ratio, efficient blood utilization, msbos, transfusion index, transfusion probability

## Abstract

Objectives

*Primary Objective*

To evaluate the efficiency of perioperative blood utilization in elective neurosurgical procedures using the crossmatch-to-transfusion (C/T) ratio, transfusion probability (TP), and transfusion index (TI).

Secondary Objective

To develop procedure-specific maximum surgical blood ordering schedules (MSBOS) using Mead’s criteria, categorizing procedures according to the need for no routine preoperative blood preparation, type-and-screen (T&S) only, or preoperative crossmatching, and to determine the recommended number of packed cell volume (PCV) units for procedures requiring crossmatching.

Materials and methods

A descriptive cross-sectional study was conducted over six months at a tertiary care hospital. A total of 142 patients (100%) undergoing elective neurosurgical procedures were included. Details of blood grouping, cross-matching, and blood transfusion were recorded for each patient. Blood utilization was assessed using the C/T ratio, TP, and TI, and the MSBOS was formulated according to Mead's criteria.

Statistical analysis

The C/T ratio, TP, and TI were calculated using standard formulae. The MSBOS was derived using the formula MSBOS = 1.5 × TI to recommend the appropriate number of preoperative blood units for elective neurosurgical procedures.

Results

Blood grouping and cross-matching were performed in 136 (95.7%) and 135 (95.4%) patients, respectively. Blood transfusion was required in 29 patients (20.4%), with 44 units transfused. The overall C/T ratio was 9.1, while the TP and TI were 22.3% and 0.33, respectively, indicating inefficient blood utilization. Relatively efficient blood utilization was observed in spinal tumour surgery, aneurysm clipping, and meningioma resection. Based on the calculated MSBOS, one unit of packed red blood cells (PRBCs) was recommended for laminectomy, spine fixation, spinal tumour surgery, aneurysm clipping, and posterior fossa surgery, whereas two units were recommended for meningioma resection.

Conclusions

Blood utilization in elective neurosurgical procedures was inefficient. Implementation of a procedure-specific MSBOS may reduce unnecessary cross-matching, optimize blood resource utilization, and improve transfusion practices in neurosurgical patients.

## Introduction

There can be a significant amount of blood loss in neurosurgical procedures, causing low tissue perfusion and risk of secondary cerebral injury requiring blood transfusion [1]. Blood is a precious and limited life-saving resource donated by members of the community; therefore, its judicious use is crucial [2]. World Health Organization reports show a huge worldwide difference in the amount of blood collected and transfused. Annual rates of blood utilization are 3.6 units per 1,000 population in low-income countries, 10.1 units per 1,000 population in middle-income countries, and 45.4 units per 1,000 population in high-income countries [3].

A study in Iran reported a utilization rate of only 38.3% of cross-matched blood in elective neurosurgeries [4]. Studies from Tanzania showed only 13.2-28.2% blood utilization in elective surgery [5,6]. Studies from North India and Ethiopia reported blood utilization rates of 22-29% [5].

The number of blood units cross-matched for a surgical procedure is usually greater than required because of worst-case assumptions. This practice adversely affects resources by reducing the shelf life of blood, limiting availability for other patients, increasing costs to both patients and institutions, and adding logistic burden [6].

On the other hand, failure to cross-match blood preoperatively may delay surgery for patients who unexpectedly require transfusion. The fundamental trade-off is between delays caused by intraoperative cross-matching and excessive preoperative cross-matching that increases costs and may lead to wastage of blood [7].

Friedman et al. derived guidelines referred to as the maximum surgical blood order schedule (MSBOS) [8]. MSBOS aims to reduce unnecessary preoperative cross-matching by recommending the maximum number of blood units that should be cross-matched for specific elective procedures [9,10].

The crossmatch-to-transfusion ratio (C/T ratio) should ideally be 1.0; however, a ratio of ≤2.5 is considered acceptable for efficient blood utilization. The transfusion index (TI), which represents the number of blood units transfused per patient cross-matched, is considered efficient when ≥0.5 [7,8]. A third parameter proposed by Mead et al., the transfusion probability (TP), is calculated by dividing the number of patients transfused by the number of patients cross-matched, with a value of ≥30% considered appropriate for routine cross-matching [7,9]. Using Mead's criteria, Subramanian et al. proposed that cross-match orders should not exceed 1.5 times the average number of blood units transfused per patient for procedures with a TP greater than 30% (blood-ordering quotient) [6,9,10].

It is generally observed that excessive preoperative cross-matching wastes human, technical, and logistic resources. There is a paucity of local research and guidelines regarding the need for cross-matching and the appropriate number of units required. Therefore, this study aimed to determine the efficiency of blood utilization in elective neurosurgical patients using the C/T ratio, TP, and TI, and to formulate a maximum surgical blood ordering schedule for specific elective neurosurgical procedures using Mead's criteria.

## Materials and methods

This is an observational cross-sectional study, conducted in the Department of Neurosurgery at a tertiary care hospital over six months. All patients above 18 years and up to 70 years of age undergoing elective neurosurgical procedures were included. Patients undergoing emergency surgery, patients less than 18 years of age, massive transfusion, febrile patients, patients with coagulopathy, with hemoglobin levels below 7, history of reaction to blood transfusions, congenital heart disease, hematological disorders such as hemophilia or thalassemia were excluded. Sample size was calculated on the OpenEpi sample size calculator. The sample size was 142. The anticipated percentage of utilization of cross-matched blood is 38.3% [2], with a level of significance of 5% and an absolute precision of 8%.

Data were collected following approval from the Ethical Review Committee, from electronic medical records and files. Variables included demographics, surgical procedure, hemoglobin levels (both pre- and postoperative levels), and blood group, number of blood units that were cross-matched, transfused and returned were collected on a structured questionnaire (Appendix). Indices such as C/T ratio, TP and TI were calculated.

Data were analysed on the IBM SPSS version 22 (IBM Corp., Armonk, NY, USA). Normality of all continuous variables was assessed by the Shapiro-Wilk test. For normally distributed data, mean and standard deviation (SD) were presented. For skewed data, median and interquartile ranges (IQR) were reported. Frequencies and percentages were calculated for categorical variables. Indices such as the C/T ratio, TP and TI, were then calculated as per definitions described above, based on which efficiency of blood utilization was assessed. For those elective neurosurgical procedures showing effective blood utilization, MSBOS was calculated. Based on the criteria mentioned above, all procedures with TP less than 5 or TI less than 0.3, neither type-and-screen (T&S) nor cross-match should be done. Procedures with a TP >5% and <30% or a TI >0.3 and <0.5 were considered suitable for T&S only. For procedures not fulfilling either of these criteria, the number of units recommended for crossmatching was calculated by multiplying the TI by 1.5.

## Results

A total of 142 patients who met the inclusion criteria were included in this study. The mean age was 44.8 ± 13.8 years. Out of 142 patients, 77 (54.7%) were males and 65 (45.2%) were females. The mean pre-operative hemoglobin level was 13± 1.8 gm/dl and the post-operative hemoglobin level was 11.8 ± 1.6 gm/dl as shown in Table 1.

**Table 1:** Descriptive statistics of age, pre- and post-operative hemoglobin levels; n=142

VARIABLE	MEAN	STANDARD DEVIATION	MIN-MAX
Age (years) n=142	44.8	±13.8	19-70
Pre-op Hemoglobin (gm/dl) n=142	13.0	±1.8	8-18
Post-op Hemoglobin (gm/dl) n=142	11.8	±1.6	7-16

The most common spinal procedures included were laminectomy (24; 16.9%), followed by spine tumors (nine; 6.3%), transpedicular screw fixation (nine; 6.3%) and microdiscectomy (seven; 4.9%). The most common cranial procedure included was supratentorial craniotomy for intra-axial lesions (29; 20.4%), followed by meningioma excision (11; 7.7%), retrosigmoid craniotomy for cerebello-pontine (CP) angle tumors (11; 7.7%), trans-sphenoidal resection of sellar lesions (nine; 6.3%), cranioplasty (eight; 5.6%), ventriculo-peritoneal shunt insertion (seven; 4.9%), posterior fossa craniotomy for other posterior fossa lesions (six; 4.2%), aneurysm clipping (six; 4.2%), deep brain stimulations (three; 2.1%) and skull lesions (two; 1.4%).

Out of a total of 142 patients, blood grouping was done. Among the patients, the most common blood group was B positive (B+), observed in 41 (30.1%) patients, followed by O positive (O+) in 39 (28.7%) and A positive (A+) in 28 (20.6%) patients. AB positive (AB+) was present in 14 (10.3%), A negative (A-) in six (4.4%), B negative (B-) in five (3.7%), O negative (O-) in two (1.5%), and AB negative (AB-) in one (0.7%) patient. Overall, the blood-group distribution accounted for all 136 patients.

Out of 136 patients whose blood group was determined, cross-match was done in 130 (91.5%) patients. In most patients (62; 47.6%), four units of packed cell volume (PCV) were arranged. Similarly, one, two, three, five, six, and nine PCVs were arranged in 15 (11.5%), 31 (23.8%), 18 (13.8%), two (1.54%), one (0.77%), and one (0.77%) patient, respectively. The total number of PCV units transfused was 44. A total of 16 (55.17%) patients received a single PCV unit while 12 (41.3%) patients received two PCV units and one (3.4%) patient received four PCV units. Description of units cross-matched and transfused is shown in Table 2.

**Table 2:** Description of units cross matched and transfused n=142

Sr. No.	Diagnosis	No. of units cross-matched, n (%)	No. of units transfused, n (%)	No. of patients cross-matched, n (%)	No. of patients transfused, n (%)
1	Laminectomy	39 (9.7)	6 (13.6)	19 (14.6)	6 (20.7)
2	Spine fixation	17 (4.2)	4 (9.1)	9 (6.9)	3 (10.3)
3	Colloid cyst	2 (0.5)	0 (0.0)	1 (0.8)	0 (0.0)
4	Intra-axial lesion	117 (29.0)	8 (18.2)	29 (22.3)	3 (10.3)
5	Ventriculoperitoneal shunt	9 (2.2)	0 (0.0)	6 (4.6)	0 (0.0)
6	Spine tumor	31 (7.7)	5 (11.4)	9 (6.9)	3 (10.3)
7	Cranioplasty	17 (4.2)	3 (6.8)	7 (5.4)	2 (6.9)
8	Meningioma	44 (10.9)	11 (25.0)	11 (8.5)	6 (20.7)
9	Sellar lesion	29 (7.2)	1 (2.3)	9 (6.9)	1 (3.4)
10	Skull lesion	4 (1.0)	0 (0.0)	1 (0.8)	0 (0.0)
11	Cerebellopontine-angle lesion	38 (9.4)	0 (0.0)	11 (8.5)	0 (0.0)
12	Discectomy	4 (1.0)	0 (0.0)	3 (2.3)	0 (0.0)
13	Aneurysm clipping	21 (5.2)	3 (6.8)	6 (4.6)	3 (10.3)
14	Posterior fossa craniotomy	22 (5.4)	3 (6.8)	6 (4.6)	2 (6.9)
15	Deep brain stimulation	10 (2.5)	0 (0.0)	3 (2.3)	0 (0.0)
Total		404 (100)	44 (100)	130 (100)	29 (100)

Out of a total of 44 PCV units, a majority (37) were transfused intra-operatively while seven were transfused post-operatively. Total number of PCV units cross-matched was 404, PCV units transfused 44, individuals transfused 29 and individuals cross-matched were 130. Overall C/T ratio was 9.2, TP 22.3% and TI 0.33. This shows that overall the utilization of blood was not efficient.

The C/T ratio for laminectomy, spine fixation, intra-axial lesion, spine tumor, cranioplasty, meningioma, sellar lesion, aneurysm clipping, and posterior fossa craniotomy was 6.5, 4.2, 14.6, 6.2, 5.7,4.0, 29, 7.0 and 7.3, respectively. Similary, TP for laminectomy, spine fixation, colloid cyst, intra-axial lesion, ventriculoperitoneal (VP) shunt, spine tumor, cranioplasty, meningioma, sellar lesion, skull lesion, CP angle lesion, discectomy, aneurysm clipping, posterior fossa craniotomy, and deep brain stimulation was 31.5, 33.3, 0, 10.3, 0, 33.3, 28.5, 54.5, 11.1, 0 ,0, 0, 50, 33.3, and 0 respectively. Similary, the TI for laminectomy, spine fixation, colloid cyst, intra-axial lesion, VP shunt, spine tumor, cranioplasty, meningioma, sellar lesion, skull lesion, CP angle lesion, discectomy, aneurysm clipping, posterior fossa craniotomy, and deep brain stimulation was 0.31, 0.44, 0.00, 0.27, 0, 0.55, 0.42, 1.00, 0.11,0.00, 0.00, 0.00, 0.50, 0.50 and 0.00 (Table 3).

**Table 3:** Blood transfusion indices n=142 C/T = crossmatch-to-transfusion, TP = transfusion probability, TI = transfusion index

Sr No	Diagnosis	C/T ratio	TP%	TI
1	Laminectomy	6.5	31.5	0.31
2	Spine fixation	4.2	33.3	0.44
3	Colloid cyst	NA	0	0.00
4	Intra-axial lesion	14.6	10.3	0.27
5	Ventriculoperitoneal-shunt	NA	0	0
6	Spine tumor	6.2	33.3	0.55
7	Cranioplasty	5.7	28.5	0.42
8	Meningioma	4.0	54.5	1.00
9	Sellar lesion	29	11.1	0.11
10	Skull lesion	NA	0	0.00
11	Cerebellopontine-angle lesion	NA	0	0.00
12	Discectomy	NA	0	0.00
13	Aneurysm Clipping	7.0	50	0.50
14	Posterior fossa craniotomy	7.3	33.3	0.50
15	Deep brain stimulation	NA	0	0.00
	Total	9.1	22.3	0.33

Based on individual procedures, laminectomy, spinal tumor surgery, aneurysm clipping and meningioma resection surgery were the only procedures with efficient utilization of blood.

Colloid cyst surgery, VP shunt insertion, skull lesions, CP angle surgery, microdiscectomy and deep brain stimulation had TP less than 5 and TI less than 0.3. So all these surgical procedures should neither have T&S nor cross-match. Supratentorial craniotomy for intra-axial lesions, cranioplasty and sellar lesions had TP between 5 and 30 or TI between 0.3 and 0.5, so all these procedures should have T&S only. Laminectomy, spine fixation, spine tumor, meningioma excision, aneurysm clipping and posterior fossa surgery had TP above 30 or TI above 0.5, so cross-match should be done in all these surgeries. For procedures not fulfilling the criteria for T&S, the number of PCV units to be crossmatched was calculated by multiplying the TI by 1.5. As blood units are available as whole units, the resulting MSBOS value was rounded up to the nearest whole unit to determine the final recommended number of PCV units for crossmatching (Table 4).

**Table 4:** Maximum Surgical Blood Ordering Schedule (MSBOS) calculation * Shows to the group which the procedure belongs based on indices TP = Transfusion Probability; TI = Transfusion Index; MSBOS = Maximum Surgical Blood Ordering Schedule; T and S = Type and Screen; T and C = Type and Crossmatch, PCV = Packed Cell Volume

Sr No	Diagnosis	TP<5 TI<0.3	TP 5-30TI 0.3-0.5	TP >30TI >0.5	MSBOS TI x1.5	Blood arrangement
1	Laminectomy	-	-	*	0.5	T and C (1 PCV)
2	Spine fixation	-	-	*	0.7	T and C (1 PCV)
3	Colloid cyst	*	-	-	-	None
4	Intra-axial lesion	-	*	-	-	T and S
5	Ventriculoperitoneal-shunt	*	-	-	-	None
6	Spine tumor	-	-	*	0.8	T and C (1 PCV)
7	Cranioplasty	-	*	-	-	T and S
8	Meningioma	-	-	*	1.5	T and C (2 PCVs)
9	Sellar lesion	-	*	-	-	T and S
10	Skull lesion	*	-	-	-	None
11	Cerebellopontine-angle lesion	*	-	-	-	None
12	Discectomy	*	-	-	-	None
13	Aneurysm Clipping	-	-	*	0.7	T and C (1 PCV)
14	Posterior fossa craniotomy	-	-	*	0.7	T and C (1 PCV)
15	Deep brainstimulation	*	-	-	-	None

## Discussion

Efficient blood utilization is essential in neurosurgical practice to minimize transfusion-related complications, optimize patient outcomes, and prevent unnecessary wastage of blood products and other limited healthcare resources. In our study, although the majority of patients underwent blood grouping (136/142, 95.7%) and cross-matching (135/142, 95.4%), overall blood utilization was found to be inefficient. The overall C/T ratio was 9.1. Among the 130 patients who underwent blood cross-matching, 29 (22.3%) required blood transfusion, yielding a TP of 22.3%, while the TI was 0.33. Most previous studies have also reported inefficient blood utilization, with overall C/T ratios of 3.44 [5], 4.9 [7], 5.83 [10], and 6.0 [4]. C/T ratios reported for individual neurosurgical procedures include 1.52 for spinal dysraphism, 1.71 for posterior fossa tumour surgeries, 14.28 for aneurysm clipping, and 1.93 for meningioma surgery [6]. In contrast, a study from Iran demonstrated efficient blood utilization, reporting a C/T ratio of 1.29 [3]. Reported TP values include 16.61% [5], 27% [7], 24.4% [10], and 15.9% [3]. Similarly, reported TI values were 0.6 [5], 0.4 [10], and 0.32 [9]. Ali et al. reported an overall C/T ratio of 3.44 in an Indian cohort [5]. Likewise, the overall C/T ratio observed in our study was substantially higher than those reported from the United Kingdom (2.11) [11], the United States (2.36) [12], and Nigeria (2.26). Ali et al. also found that spinal dysraphism had the most favourable C/T ratio (1.52), followed by posterior fossa tumours and meningiomas, whereas considerable blood wastage was observed in patients undergoing surgery for subarachnoid haemorrhage (C/T ratio 14.2) and pituitary tumours (10.12) [5].

Among the 130 patients who underwent blood cross-matching, 29 (22.3%) required blood transfusion. Ali et al. reported an overall TP of 16.61%, indicating inappropriate utilization of blood products [5]. Based on TP, spine fixation surgery, spinal tumour surgery, meningioma excision, and aneurysm surgery in our cohort demonstrated relatively efficient blood utilization. Ali et al. further reported that patients with spinal dysraphism (42.70%) had the highest probability of requiring blood transfusion, followed by posterior fossa tumours (33.33%), craniovertebral junction anomalies (33.33%), and meningiomas (28.57%). Conversely, patients with subarachnoid haemorrhage (3.00%) and pituitary tumours (6.17%) had the lowest TP [5]. These findings were comparable to those reported by Belayneh et al. [13].

The overall TI in our study was 0.33. Among individual procedures, laminectomy for spinal tumours, aneurysm clipping, and meningioma resection were the only procedures demonstrating efficient blood utilization. In the study by Ali et al., a TI greater than 0.5 was observed in patients undergoing surgery for spinal dysraphism, posterior fossa tumours, and craniovertebral junction anomalies. Accordingly, the highest MSBOS recommendations were for spinal dysraphism, followed by posterior fossa tumours, meningiomas, and craniovertebral junction anomalies [5].

Similarly, another local study evaluating blood-ordering practices across various surgical specialties reported a C/T ratio of 1.7 and a TI of 0.4 for neurosurgical procedures [14]. However, local data on this topic remain scarce. Chawla et al. conducted one of the earliest audits in 2001, evaluating blood cross-matching practices across multiple surgical specialties. Among neurosurgical procedures, lumbar disc surgery demonstrated the highest mean C/T ratio (82). Based on these findings, the authors recommended a group-and-screen (G&S) policy rather than routine cross-matching for this procedure [15].

A study evaluating preoperative blood-ordering practices in elective spinal fusion surgery found that, of the 292 packed red blood cell (PRBC) units ordered for 85 patients, only 66 units (22.6%) were transfused, resulting in a high C/T ratio of 4.4:1. The highest C/T ratios were observed in cervical arthrodesis (23:1) and degenerative spinal disease (6.9:1). The authors recommended reserving two units of PRBCs only for patients with haemoglobin <9 g/dL, trauma, adolescent idiopathic scoliosis (AIS), or multilevel procedures, while a group-and-hold policy was considered sufficient for all other patients [16]. A subsequent validation study demonstrated that implementation of this protocol reduced the C/T ratio from 4.02 to 1.74 and decreased per-patient costs by 30% (PKR 6676.8 to 4700.8; P < 0.001) [17].

These findings underscore the need for improved blood management strategies in neurosurgical practice, including the implementation of evidence-based transfusion protocols and blood conservation measures. The procedure-specific MSBOS developed in the present study provides practical recommendations for preoperative blood ordering and has the potential to reduce unnecessary cross-matching, conserve blood products, and improve resource utilization without compromising patient safety. The proposed MSBOS may help optimize preoperative blood ordering and reduce unnecessary crossmatching in elective neurosurgical procedures based on observed transfusion patterns. Prospective validation is warranted before routine implementation to assess its impact on blood availability, transfusion requirements, and patient safety. It will improve resource allocation, minimize unnecessary transfusions, and reduce healthcare costs [18]. Furthermore, prospective studies evaluating standardized blood-ordering protocols and their clinical impact are warranted to validate these findings and strengthen evidence-based blood management practices in neurosurgery.

This study has several limitations. First, its single-center observational design and relatively small sample sizes for some procedure-specific groups may limit the generalizability of the findings. Second, although blood-utilization data were prospectively reviewed, the proposed MSBOS was derived from observed transfusion patterns and was not prospectively implemented or validated. Third, transfusion requirements may have been influenced by procedure complexity, intraoperative blood loss, surgeon preference, patient comorbidities, and institutional transfusion practices, which were not fully controlled for in this descriptive analysis. In addition, the study did not evaluate emergency blood availability, transfusion delays, operative cancellations, transfusion-related adverse events, mortality, or whether patients required blood beyond the proposed MSBOS. Therefore, the proposed schedules should be considered preliminary and hypothesis-generating rather than definitive recommendations. Multicenter prospective studies with implementation and safety outcomes are required to validate these procedure-specific MSBOS recommendations before routine clinical adoption.

## Conclusions

Despite variations in TP and TI across procedures, the findings demonstrate substantial differences in perioperative blood utilization among elective neurosurgical procedures. Based on the observed transfusion patterns and predefined MSBOS criteria, colloid cyst surgery, VP shunt insertion, skull lesions, CP angle surgery, microdiscectomy, and deep brain stimulation may be managed without routine preoperative T&S or crossmatching. Supratentorial craniotomy for intra-axial lesions, cranioplasty, and sellar lesions may require T&S only. Laminectomy, spine fixation, spinal tumor surgery, meningioma excision, aneurysm clipping, and posterior fossa surgery may warrant preoperative crossmatching, with one PCV unit recommended for all except meningioma excision, for which two PCV units are recommended. These procedure-specific recommendations may help optimize blood ordering and reduce unnecessary crossmatching; however, prospective validation is required before routine implementation and to assess their safety in clinical practice.

## Appendices

QUESTIONNAIRE

Serial No. ----------------------------

Study ID No.       ----------------------------

AGE in years        -----------------------------

GENDER        ---------------------------------

PROCEDURE---------------------------------- 

DIAGNOSIS -----------------------------

Blood group--------------------

BLOOD GROUP AND HOLD ONLY    1. YES     2. NO

BLOOD CROSS MATCHED     1. YES     2. N0

Number of units cross-matched ---------------------- 

Number of units transfused-----------------------------

Number of units transfused intraoperative----------------------

Number of units transfused postoperative --------------------------

Pre-op Hemoglobin (gm./dl) ----------------------------

Post-op Hemoglobin (gm./dl) ---------------------------

MSBOS ---------------------------------
